# Medical History from the Earliest Times

**Published:** 1892-06-25

**Authors:** E. T. Withington


### Medical History from the Earliest Times.

By E. T. Withington, M.A., M.B. Oxon.

### XIV.—HERACLIDES AND THE EMPIRIC SCHOOL.

Some readers may have found the name Heraclides, mentioned among typical Greek physicians in a preceding article, rather strange to them, but in Heraclides of Tarentum, Heraclides the Empiric (b.c. 230), we may, I think, find an almost forgotten hero of medicine, some of whose claims to remembrance shall here be briefly repeated. His works have perished—our relics of ancient literature are by no means survivals of all the fittest—and what we know of him is gathered from scattered notices in later writers. Galen calls him "a most excellent physician," giving him the high praise that he never preferred his party to the truth, and Soranus considered him the only Empiric worth refuting; had he not been in his grave for three centuries, he might have given the great Methodist something more to do. But Heraclides was not fond of controversy, and his lukewarmness in that respect led him to be accused of relapsing into dogmatism. He did not, like Serapion, seek notoriety by abusing his colleagues, or by introducing some new and startling remedy which should excel the virtues of tortoise blood or crocodile dung, but devoted himself to the humbler task of weeding the already over-luxuriant garden of Empiric medicines. This he did in his greatest work "On the Preparation and Proving of Drugs," which he declared, in the best spirit of Empiricism, contained nothing but what he had himself observed, and which formed a rich mine for all future writers on *materia medica*. In this treatise Heraclides seems to have first pointed out the great value of opium, and to have defined the indications for its use. That drug, indeed, was, as we have seen, probably used from the earliest times, and though only once mentioned in the Hippocratic writings, was employed by Diocles (b.c. 350) as a remedy for toothache, and is noticed by his contemporary Diagoras, who asserts that it acts injuriously on the special senses, and is, therefore, to be avoided in affections of the eye and ear. Heraclides, however, disregards this, and recommends poultices containing the drug in painful ophthalmia. We have also prescriptions of his containing opium for cases of sleeplessness, spasm, cough, "cholera," and colic, and one with a large dose of the drug for patients bitten by venomous serpents. His treatment of "brain fever," or phrenitis, of which he distinguished a cerebral and gastric form, was much praised, and consisted of a darkened room, cold to the head, bleeding, and enemata; while in opposition to the dominant school, he declared that in acute fevers fluids are not to be withheld from the thirsty sufferer. He was scarcely less distinguished as a surgeon, asserted the possibility of reducing dislocations of the hip, and invented a machine for that purpose; indeed it seems highly probable that it was Heraclides who first conceived the idea of utilising the mechanical inventions of his contemporary and neighbour, Archimedes, in surgery. He also invented a method for separating the eyelid from the ball, when adherent after injury, which was practised for many centuries. Two works of more general interest, "On Cosmetics," and "On Diet in Health" (Symposium), are attributed to Heraclides, both of which are said to have been the first of their kind. From the former Galen has extracted two recipes "for making the hair stick together," the first consisting of wax, pitch, glue, and gum-mastich, equal parts, to be warmed before using. Did the ancient Greeks wax their moustaches? For incipient baldness, Heraclides recommends a pomade of anemones rubbed up in oil, which he says will also darken the hair. Among the scanty extracts which survive from the Symposium, we may notice the assertion that sheep's trotters, snails, and other glutinous substances cause indigestion if taken in excess, and that it is always well to eat a little before drinking. Finally, this prolific author wrote commentaries on several Hippocratic works, where, in contrast to other Empirics, he showed due respect to the memory of the mighty Asclepiad, and he has received as an appropriate reward the honourable mention of his successors of every school.

To pass to the sect of which Heraclides was the greatest ornament; Empiricism is now a by-word and a reproach in medicine, but the system originally so called owed its origin mainly to the teaching of one whose name on the great roll of the Asclepiadæ precedes even that of Hippocrates, the most scientific of the Greeks, the grandest intellect, perhaps, of the human race. Aristotle, son of the Asclepiad Nicomachus, probably studied medicine in early youth, and may even have practised it; his anatomical discoveries are worthy to be compared with those of Herophilus and Erasistratus, and his teaching, which brought down the philosophy of Plato from heaven to earth, from the ideal to the real, made it at least more suited for that art which deals with human bodies. As the result, partly of this teaching, partly of the sceptic philosophy taught about the same time by Pyrrho, and partly of the natural reaction against the extravagant theorising of the Dogmatists, there arose at Alexandria, about 280 b.c., the Empiric school of medicine, of which the more direct founders were two pupils of Herophilus, Philinus and Serapion. Of these the former argued with the Dogmatists, and the latter abused them, not sparing even Hippocrates himself. Indeed, Serapion seems to have been a sort of Greek Paracelsus, and, like the notorious German, is said to have introduced an important mineral remedy into medicine, and to have first used sulphur in chronic skin diseases. The Empirics, though a branch of the Alexandrine School, despised anatomy. They even wrote treatises to prove its worthlessness, and we may well fancy Serapion exclaiming, in the language of Paracelsus, "What is the use of knowing the shape and position of the brain and liver, or whether there are such things as brains and livers at all?" No less did they reject the Dogmatic physiology and pathology, and one of their favourite mottoes was, "It is not the cause, but the cure of diseases that concerns us; not how we digest, but what is digestible." In short, they reduced the whole art and science of medicine to a system of therapeutics. A person is ill, that is, he has certain unpleasant feelings or symptoms; surely the first thing to do is to find something which will remove them, and the whole duty of the physician is to discover what particular treatment, and especially what drugs, will get rid of particular sets of symptoms. This he may do in three ways: (1) By his own observations and experiments—autopsy; (2) by learning from his contemporaries and predecessors—history; (3) in the case of new and strange diseases, by drawing conclusions from those most similar to them—analogy. Thus was established the famous "tripod" of the Empirics, but being found rather shaky on its three legs, a fourth was afterwards added, "epilogism," or the process of inferring preceding events from the present symptoms. Thus, by epilogism, the consistent Empiric might conclude from the extreme inflammation of a wound that it was poisoned, and treat it accordingly without falling into the Dogmatic heresy of looking for hidden causes. Empiricism practically resolved itself into a search for specifics, and its immediate result was the introduction of a great number of drugs, some of very extraordinary nature, such as hare's heart, camel's brain, the flesh of weasles, and of human beings, and the two examples given above; but doubtless there was ample evidence, both from "autopsy" and "history," of patients recovering after taking any of them.

It has been said that the besetting sin of men of science is to fancy they have finished off all things in heaven and earth by giving them names. The Empirics, says Galen, were "terrible men for names," and in this they were encouraged by Aristotle, who was not only the father of natural science, but had other offspring, one of whom sprang from his marvellous brain a full-grown Pallas Athene. This was Logic, and its definitions and syllogisms were seized upon with delight by the physicians of the day, who soon showed that as much mental energy could be wasted in word-splitting, definitions of the pulse, &c., as in the vaguer speculations of Dogmatism.

The Empirics existed as a separate school for some centuries, and we shall find one of them among the teachers of Galen; but they finally separated into two distinct branches, one philosophical, and culminating in the sceptic agnosticism of Sextus Empiricus'; while the other, or practical branch, gradually degenerated into Empiricism in its modern sense, and found its chief exponent in Marcellus the Empiric, in whose writings human credulity in matters medical seems to have achieved its utmost. Curiously enough, these two are almost the only members of the sect of whose works we possess more than fragments, and they may be considered in greater detail later on.

Meanwhile, let us remember that the Empiric school was useful in its generation in checking the extravagances of Dogmatism, and in extending and defining the use of such remedies as opium and sulphur, and, above all, that it produced one great physician worthy to stand with the noblest round the Hippocratic throne, Heraclides of Tarentum.

